# Artificial intelligence and machine learning in clinical development: a translational perspective

**DOI:** 10.1038/s41746-019-0148-3

**Published:** 2019-07-26

**Authors:** Pratik Shah, Francis Kendall, Sean Khozin, Ryan Goosen, Jianying Hu, Jason Laramie, Michael Ringel, Nicholas Schork

**Affiliations:** 1 Massachusetts Institute of Technology, Media Laboratory, Cambridge, MA USA; 2 F. Hoffmann-La Roche AG, Strategic Innovation, San Francisco, CA USA; 3 US Food and Drug Administration, Silver Spring, MD USA; 4 The Boston Consulting Group, Boston, MA USA; 5 IBM Research, Center for Computational Health, New York, NY USA; 6 Novartis Institute of Biomedical Research, Cambridge, MA USA; 7 The Translational Genomics Research Institute, Quantitative Medicine and Systems Biology Phoenix, Phoenix, AZ USA

**Keywords:** Translational research, Computer science

## Abstract

Future of clinical development is on the verge of a major transformation due to convergence of large new digital data sources, computing power to identify clinically meaningful patterns in the data using efficient artificial intelligence and machine-learning algorithms, and regulators embracing this change through new collaborations. This perspective summarizes insights, recent developments, and recommendations for infusing actionable computational evidence into clinical development and health care from academy, biotechnology industry, nonprofit foundations, regulators, and technology corporations. Analysis and learning from publically available biomedical and clinical trial data sets, real-world evidence from sensors, and health records by machine-learning architectures are discussed. Strategies for modernizing the clinical development process by integration of AI- and ML-based digital methods and secure computing technologies through recently announced regulatory pathways at the United States Food and Drug Administration are outlined. We conclude by discussing applications and impact of digital algorithmic evidence to improve medical care for patients.

## Introduction

Clinical drug development has remained relatively unchanged for the last 30 years. This is due, in part, to uncertainties in regulatory requirements, risk aversion, and skepticism about rapidly emerging, yet largely unproven, technologies (such as machine learning, and wireless health monitoring devices and sensors), and the lack of relevant actionable biomedical data sources and advanced analytics to generate hypotheses that could motivate the development of innovative diagnostics and therapies. Testing new biomedical treatments for safety and efficacy will also require new strategies, since it has been shown that existing therapies often only work for a small number of indicated individuals. The application of emerging digital technologies, such as next-generation sequencing, though, have increased both our understanding of disease mechanisms in larger pool of patients and the potential for developing personalized therapies. For example, the majority of the new molecular entities approved by the U.S. FDA in recent years were designed to target specific aberrations implicated in disease initiation and maintenance—a hallmark of precision medicine—which aims to tailor interventions based on individual characteristics of patients.^1^ In this light, an emerging strategy based on co-developing precision diagnostics and therapeutic agents as companion diagnostics for example may produce highly effective drugs with clinical outcomes that greatly exceed standard therapies.^2,3^

Another key challenge in the clinical development process is linked to reporting the results of most conventional clinical trials of average treatment effects that may not easily translate into making individualized treatment decisions at the routine point-of-care.^4^ Promising approaches to overcoming this challenge are more streamlined processes, exploiting new digital clinical endpoints and treatment response biomarkers amenable to close and efficient monitoring (such as circulating tumor DNA), improve safety and efficacy while reducing toxicity and adverse events and greater insights into the patient journey via sensors, and low cost imaging.^5–8^ Securing, standardizing, and enhancing routinely collected EHR data as a source of credible medical evidence based on RWD can facilitate the organization of clinical trials at the point-of-care and should serve to improve the clinical development process.^9^

Machine learning and computer vision have enhanced many aspects of human visual perception to identify clinically meaningful patterns in, e.g., imaging data,^10^ and neural networks are been used for variety of tasks ranging from medical image segmentation, generation, classification, and prediction of clinical data sets.^11^ Broadly academic research labs, biotechnology corporations, and technology companies have been exploring the use of AI and ML in three key areas:

1. machine-based learning to predict pharmaceutical properties of molecular compounds and targets for drug discovery;^12,13^
2. using pattern recognition and segmentation techniques on medical images (from, e.g., retinal scans, pathology slides and body surfaces, bones and internal organs) to enable faster diagnoses and tracking of disease progression;^14,15^ and generative algorithms for computational augmentation of existing clinical and imaging data sets;^16^
3. developing deep-learning techniques on multimodal data sources such as combining genomic and clinical data to detect new predictive models.^17,18^

Despite these propositions for the use of ML to accelerate medical research, very few successful use cases have emerged. These limited successes have been attributed to, among other things, insufficient time elapsing since the introduction of relevant technologies and deficiency of current computer science deep learning and related ML models to generalize more complex and realistic medical data sets and tasks.^19,20^ Other important factors that impede the adoption of AI/ML techniques in therapeutic development include the paucity of large numbers of high-quality labeled data, nascent regulations, and ethical and legal concerns about data sharing. Alternative learning systems that leverage human brain and its neocortex and learn from fewer examples have been proposed as alternatives to deep learning, but have not been widely adopted.^21^ Recently, perspectives and commentaries highlighting applications of DNN to imaging data sets, pharmaceutical properties of compounds, clinical diagnoses and genomics, computer vision applications for medical imaging, and applications of Natural Language Processing to EHR have been published.^22,23^ These predominantly focused on data in primary care or hospital ecosystem and early drug discovery applications, and did not describe use cases and regulatory framework derived from a multi-stakeholder perspective for successful embedding of AI and ML and RWE into the process of clinical development outlined in this perspective.

From March 2017 to December 2018, a series of six broad, cross-institutional workshops were convened at The MIT Media Lab to discuss the current state of AI and ML and RWE usage in clinical development opportunities, challenges, and ways of addressing challenges. Participation was designed to be multidisciplinary and multi-stakeholder, involving leading researchers from academic institutions, leaders from biopharma firms, foundations technology corporations, and regulators to engender broad outlook and cross-functional perspectives. Each two-part workshop was structured as follows: a series of talks outlining current challenges and opportunities and regulatory insights for introducing AI and ML in the clinical development process either as researchers or adopters, followed by a brainstorming session with breakaway groups focusing on specific themes. This manuscript, a consolidated viewpoint on infusion of AI and ML in clinical development, is one of the key outputs of the workshop. We focus on three key themes discussed in the workshops related to development of next-generation medicines by adoption of digital evidence generated by AI and ML: (1) validation and modernizing the clinical trials process, (2) strategies for rational use of AI- and ML-driven learning from real-world data and evidence and, (3) required regulatory oversight for integration, explanation, and de-risking of AI/ML digital analytics in medical care to patients. A glossary is provided as Supplementary Material for explanation of key terms.

## Discussion

MIT Workshops discussed new pathways set up by regulatory agencies for evaluation and adoption of AI and ML in clinical development. For example, in 2016, the 21^st^ Century Cures Act was signed into law, a significant bipartisan legislative achievement aimed at accelerating the discovery, development, and delivery of new cures and treatments was highlighted. FDA’s current strategic policy places emphasis on leveraging innovation, advancing digital health technologies, and developing next-generation analytical approaches to improve health care, broaden access, and advance public health goals. SaMD and digital health pathways for regulatory approvals for AI, ML, and computer vision algorithms have been set up at FDA.^24,25^ To date, FDA has cleared or approved several AI/ML-based SaMD. Typically, these have only included algorithms that are locked prior to marketing. For example, FDA has approved diagnostics company IDx’s ML-based software system for autonomous detection of diabetic retinopathy.^26^ In addition, Viz.ai’s software, which uses a ML techniques to scan Computed Tomography images for indicators associated with stroke, also obtained the regulatory approval.^27^ Other software systems listed included automated detection of atrial fibrillation and coronary calcification scores.^28,29^ The FDA is also considering the ability of AI/ML-based SaMD for continuously learning and adaptive algorithms that have the potential to adapt and optimize device performance in real time to improve health care for patients in its regulatory framework. More broadly, FDA is using significance of information provided by SaMD to health care decision such as treat or diagnose, drive clinical or inform clinical management as key determinations for regulatory strategies. Pharmaceutical, biotechnology, and startup community participating in the workshops has recognized the potential of AI and ML in the development of personalized medicines and generating evidence motivating new products (summarized in Box 1). As examples, Johnson & Johnson Innovation’s life sciences “JLABS” newco incubator currently includes ML startups, such as Analytics 4 Life, A2A Pharmaceuticals, Envisagenics, among others. Precision medicine company GNS Healthcare and Genentech aim to collaboratively discover and validate new oncology drugs and patient response markers. Pfizer and Novartis are each working with IBM Watson Health to facilitate immuno-oncological research and development. Glaxosmithkline recently announced the creation of “Accelerating Therapeutics for Opportunities in Medicine (ATOM)” Consortium to accelerate drug discovery process using ML tools.^30^ These early initiatives are either set up as in-house research groups or as public–private partnerships to engender cross-functional teams.

Discussions also focused on RWD-based simulations are now accepted as a reasonable way to inform clinical study design, modeling the impact of different study eligibility criteria, the timing of endpoint assessments, and study timelines at the FDA.^31^ Treatment and regulatory decisions are based largely on data obtained from clinical trials and observational real-world data, and evidence from hospitals, EHR, and primary care are considered ancillary. An approach for evaluating RWE for clinical development and regulatory decisions would have the advantage of getting the medicine to the patient more quickly, with a better and fuller understanding of how effective and safe the medicines are in real-world settings. RWD can also inform study site selection, as well as identify patients potentially eligible for trials. Simulation of study control arms, potentially replacing the need to randomize a patient to a control arm in some scenarios, is another successful use of RWD. Authors note emergence and impact of analytics of RWD and RWE in academic biomedical science and health care delivery. For example, large “data lakes” have been created by aggregating information from hospital EHR, creating an opportunity for exploration via AI and ML approaches to identify clinically meaningful patterns.^32–34^ These data lakes can be used to and ability to track patients longitudinally. By using RWD and tracking patients longitudinally via EHR for example, necessity for certain traditionally conducted late-phase trials could be reduced or eliminated altogether: one would provide a drug (after it has proven safe and efficacious in phase I and II trials) to patients and keep track of their experience.

Another theme at workshops focused on novel trials designs such as “basket,” “umbrella,” and many adaptive designs have been encouraged by regulatory agencies and can exploit emerging AI and ML techniques. These designs can enroll patients in a trial, profile them (e.g., using DNA sequencing, proteomics, metabolomics, etc.), and then use RWD for matching drugs considered in the trial to the pathologies identified from the profiling. Strategies for matching drugs to patient profiles in these studies can be based on AI and ML analysis of large relevant data sets. AI and ML can further be used to support an electronic version of study data monitoring, thereby ensuring that data are correct and the patients are safe; thus reducing the need for expensive on-site study monitoring. Furthermore, EHR data can be combined with other RWD types, such as genomics and patient-reported concerns, can be mined with AI and ML techniques to create a more comprehensive picture for drug and biomarker discovery. As methods for each of these tasks are determined and refined, computational solutions, including AI and ML, can be implemented to reliably replicate clinical trial activities at scale. These types of clinical trials—which ultimately test intervention “algorithms” such as drug–patient profile matching schemes—are likely to become more pronounced and prevalent in the future, and could be greatly facilitated by leveraging clinical outcomes monitoring and RWD collection. This interaction could also lead to the development of “Clinical Decision Support” tools that provide insight into optimal ways of treating patients. Experience with these tools could lead to further refinements, ultimately providing continuous feedback on their effectiveness.

**Fig. 1:**
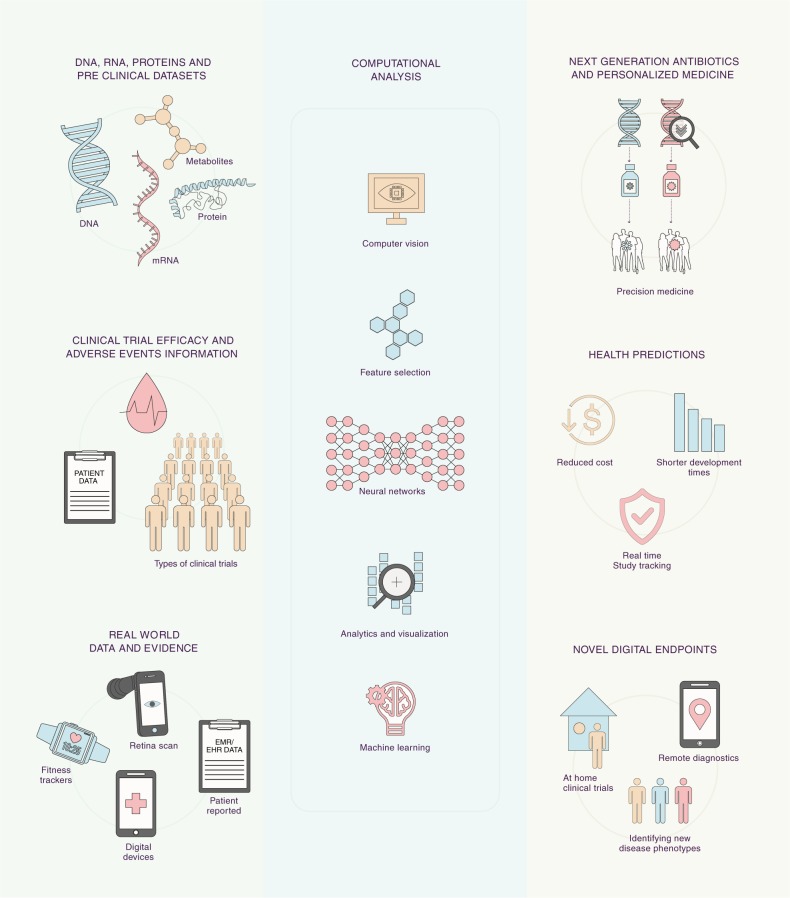
Use cases of artificial intelligence, computer vision, and machine learning in clinical development

Recent research at the intersection of computer science and medicine, proactive regulatory landscape, and availability of large data sets offers use cases and promise of testing and delivering faster cures to patients by leveraging sophisticated AI and ML methods (Fig. 1). This perspective aims to engage and inform researchers from fields, such as computer science, biology, medicine, engineering, biostatistics, and policy makers, with value of emerging technologies of AI and ML in solving key challenges facing modernization of the current clinical development process. Accordingly, Box 1 lists specific call-to-action, use cases, and considerations for different stakeholders across biotechnology and technology companies, foundations, regulators, and academic institutions discussed in a series of workshops. The authors also note that collaborations between engineering, medical imaging, machine learning, secure computing, and medicine have recently been fostered at various academic institutions across United States showing commitment toward such efforts. For example, MIT Media Lab and United States FDA signed a Memorandum of Understanding “Health 0.0” to engender AI and ML research for computational medicine and clinical development and accompanying regulatory framework to improve health outcomes for patients. Life sciences, biotechnology, foundations, universities, and patient advocacy groups are parts of this ecosystem. Partnerships at MIT with IBM, Abdul Latif Jameel Foundation Clinic, and Stephen A. Schwarzman College of Computing have been launched to support machine-learning research for health care needs.^35,36^ Partnership in AI-assisted care at Stanford, Center for Artificial Intelligence in Diagnostic Medicine at University of California, Irvine and Center for Clinical Data Sciences at Massachusetts General Hospital and Brigham and Womens Hospital have joined the ecosystem.^37–39^ A number of key recommendations and successful use cases and value and challenges facing AI and ML adoption in clinical development outlined in this perspective (summarized in Box I) can thus be implemented for taking advantage of digital algorithmic evidence to improve medical care for patients.

Box 1. Key Recommendations, action items, and challenges **Biotechnology industry**

- Maximize AI and ML opportunities by focusing on data aggregation and easier access to data within and across pharma, biotech, and infotech companies.
- Shared data in a noncompetitive manner (such as, e.g., TransCelerate placebo data, DREAM challenge and IBM, Project DataSphere) with multiple parties in the health care ecosystem, including technologists and academics to generate insights faster and collaboratively.^40–43^
- Embed computer science experts in relevant initiatives in order to build core capabilities and interdisciplinary teams, as well as leverage advisors from academic research groups.

**Academy**

- New research, and academic and education departments focusing on training next generation of AI and ML professionals with experience in learning from clinical data.
- Develop ML and AI algorithms capable of continuous learning from multimodal and sparse input data usually found in clinical development.
- Host high value data sets in a secure public–private–government sandbox to engender research to infuse AI and ML in clinical development and design new digital medicine tools with an eye toward facilitating collaborations.^44^
- Develop AI ML toolboxes that are publically available for use by multiple stakeholders.^45^

**Regulatory agencies**

- Consider AI and ML techniques as effective tools and aids to drug development.^46^
- Evaluate the use of real-world evidence, generated by data outside traditional clinical trials from sources such as electronic health records and digital health devices, to support new drugs and health technologies.^47^
- Expand and promote internal innovations groups to engage actively with academic institutions, other government agencies, and technology companies to improve the clinical development process.^48^

**Technology corporations**

- Promote and maintain collaborations with academic groups developing AI and ML tools as such collaborations can also be used to shape the creation of a “future” workforce that can have a bigger impact on health care.
- Leverage expertise across the whole spectrum of computing: from hardware designs, including quantum computing, to security, platforms, and services, in order to develop platforms for efficient and agile development.

**Challenges and considerations**

- Limitations of current computer science deep learning models to generalize to complex medical data sets and tasks.
- Necessities of high volumes of labeled data sets for training deep learning algorithms
- Strategies and regulatory framework for dealing with relevant ethics issues (e.g., patient privacy, retaining anonymity, securing data)^45^ and de-risk use of AI- and ML-based clinical prediction and decision support in health care.^49^

## Supplementary information

Supplementary information

## Supplementary information

**Supplementary Information** accompanies the paper on the *npj Digital Medicine* website (10.1038/s41746-019-0148-3).
