# Supplementary material for: Artificial intelligence and machine learning in clinical development: a translational perspective
Source: NPJ Digit Med. 2019 Jul 26;2:69. doi: 10.1038/s41746-019-0148-3 (PMC6659652; doi:10.1038/s41746-019-0148-3)
Supplement: Supplementary file 1 — Supplementary information [file 41746_2019_148_MOESM1_ESM.pdf]

Supplementary Material

Glossary

Artificial Intelligence: **AI,** An area of computer science that deals with giving machines the ability to seem like they have human intelligence.

Clinical Development: **CD**, is a blanket term used to define the entire process of bringing a new drug or device to the market. It includes drug discovery/product development, pre-clinical research (microorganisms/animals) and clinical trials (on humans).

Deep Neural Network: **DNN**, is an artificial neural network with multiple layers between the input and output layers. The DNN finds the correct mathematical manipulation to turn the input into the output, whether it be a linear relationship or a non-linear relationship.

Electronic Health Record: **EHR**, is a longitudinal electronic record of patient health information generated by one or more encounters in any care delivery setting. Included in this information are patient demographics, progress notes, problems, medications, and vital signs, past medical history, immunizations, laboratory data and radiology reports.

Food & Drug Administration: **FDA**

Machine Learning: **ML**, is the scientific study of algorithms and statistical models that computer systems use in order to perform a specific task effectively without using explicit instructions, relying on patterns and inference instead. It is seen as a subset of AI.

Real World Data: **RWD**, are the data relating to patient health status and/or the delivery of health care routinely collected from a variety of sources. RWD can come from a number of sources, for example: EHRs, Claims and billing activities, Product and disease registries, Patient-generated data including in home-use settings, Data gathered from other sources that can inform on health status, such as mobile devices

Real World Evidence: **RWE**, is the clinical evidence regarding the usage and potential benefits or risks of a medical product derived from analysis of RWD

Software as a Medical Device: **SaMD**, software intended to be used for one or more medical purposes that perform these purposes without being part of a hardware medical device.
